# Communication transforms the impact of the COVID‐19 pandemic on children with cancer and their families

**DOI:** 10.1002/cam4.5950

**Published:** 2023-04-20

**Authors:** Gia Ferrara, Molly Aguina, Emily Mirochnick, Parima Wiphatphumiprates, Daniel C. Moreira, Elizabeth Sniderman, César A. Villegas, Erica C. Kaye, Iman Ragab, Biemba Maliti, Gita Naidu, Pascale Y. Gassant, Daniela Arce, Ramandeep Singh Arora, Ana Patricia Alcasabas, Muhammad Rafie Raza, Pablo Velasco, Joyce Kambugu, Anna Vinitsky, Carlos Rodriguez Galindo, Asya Agulnik, Dylan E. Graetz, and the COVIMPACT study group, Aliaksandra Laptsevich, Haiying Huang, Luciana Nunes Silva, Jacqueline Montoya Vasquez, Desy Rusmawatiningtyas, Nancy S. Bolous, Cyrine E. Haidar, Laure Bihannic, Diana Sa da da Bandeira, Jade Xiaoqing Wang, Dongfang Li, Flavia Graca, Aksana Vasilyeva, Harry Lesmana

**Affiliations:** ^1^ St. Jude Children's Research Hospital Memphis Tennessee USA; ^2^ Princeton University Princeton New Jersey USA; ^3^ The Chicago Medical School at Rosalind Franklin University of Medicine and Science North Chicago Illinois USA; ^4^ Rhodes College Memphis Tennessee USA; ^5^ Northern Alberta Children's Cancer Program Stollery Children's Hospital Edmonton Alberta Canada; ^6^ Ain Shams University, Children's Hospital, Hematology‐Oncology Unit Cairo Egypt; ^7^ Cancer Diseases Hospital Lusaka Zambia; ^8^ Chris Hani Baragwanath Academic Hospital University of the Witwatersrand Johannesburg South Africa; ^9^ Hospital Saint‐Damien Port‐au‐Prince Haiti; ^10^ Hospital Pediátrico de Sinaloa Culiacan Mexico; ^11^ Max Super Specialty Hospital New Delhi India; ^12^ University of the Philippines Philippine General Hospital Manila Philippines; ^13^ The Indus Hospital Karachi Pakistan; ^14^ Pediatric Oncology and Hematology Department Vall d'Hebron Hospital Barcelona Spain; ^15^ Uganda Cancer Institute Kampala Uganda

**Keywords:** clinical cancer research, pediatric cancer, psychosocial studies, quality of life

## Abstract

**Background:**

The COVID‐19 pandemic altered healthcare systems globally, causing delays in care delivery and increased anxiety among patients and families. This study examined how hospital stakeholders and clinicians perceived the global impact of the COVID‐19 pandemic on children with cancer and their families.

**Methods:**

This secondary analysis examined data from a qualitative study consisting of 19 focus groups conducted in 8 languages throughout 16 countries. A codebook was developed with novel codes derived inductively from transcript review. In‐depth analysis focused on the impact of the COVID‐19 pandemic on children with cancer and their families.

**Results:**

Eight themes describing the impact of the pandemic on patients and their families were identified and classified into three domains: contributing factors (*COVID‐19 Policies, Cancer Treatment Modifications, COVID‐19 Symptoms, Beliefs*), patient‐related impacts (*Quality of Care, Psychosocial impacts, Treatment Reluctance*), and the central transformer (*Communication*). Participants described the ability of *communication* to transform the effect of contributing factors on patient‐related impacts. The valence of impacts depended on the quality and quantity of communication among clinicians and between clinicians and patients and families.

**Conclusions:**

Communication served as the central factor impacting whether the COVID‐19 pandemic positively or negatively affected children with cancer and families. These findings emphasize the key role communication plays in delivering patient‐centered care and can guide future development of communication‐centered interventions globally.

## Graphical abstract

This multinational, qualitative secondary analysis demonstrates how communication transformed the effects of the COVID‐19 pandemic on patients and families. The findings of this study emphasize the importance of developing communication centered interventions as a means of mitigating impacts on patient care.

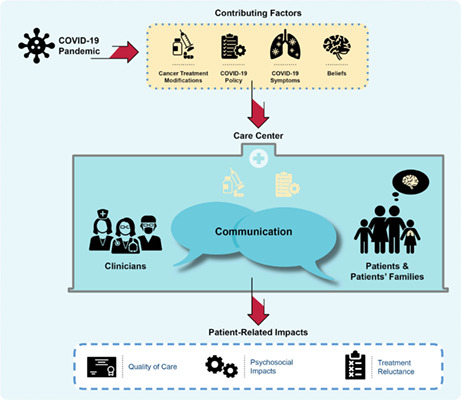

## 1 INTRODUCTION

The coronavirus disease 2019 (COVID‐19) pandemic impacted healthcare globally and exacerbated existing healthcare systems weaknesses. Many countries faced shortages in personal protective equipment, medications, and workforce,^1^, ^2^, ^3^ with effects greatest in low‐ and middle‐income countries (LMICs).^4^, ^5^ The burden of the pandemic was disproportionately higher in LMICs with people in lower‐income settings having a much higher infection fatality rate by age compared to their counterparts in higher‐income settings^6^ and resource shortages being more highly exacerbated in LMICs.^7^ For children with cancer, the COVID‐19 pandemic caused delays in diagnosis^8^; interrupted cancer‐directed treatment^9^, ^10^, ^11^, ^12^; reduced supportive care,^13^ hospital visits,^14^ and new patient admissions.^4^, ^15^, ^16^ Potential interruptions in treatment and canceled hospital visits, in addition to general risk of infection from COVID‐19, intensified an already present psychological burden experienced by children with cancer and their families leading to an increased risk for post‐traumatic symptoms in parents of children with cancer and high levels of stress and anxiety.^17^, ^18^

Due to immunosuppression caused by cancer‐directed treatments, children with cancer are particularly vulnerable to SARS‐CoV‐2 infection.^19^ To cope with changes caused by the pandemic and limit patient exposure to the virus, hospitals focused on telehealth and implemented masking and social distancing mandates.^20^, ^21^

To date, many research studies have primarily focused on the quantitative impacts of the COVID‐19 pandemic on childhood cancer care delivery regionally or by country.^4^, ^22^ There is limited literature regarding the pandemic's effect on children with cancer and their families across diverse settings globally. In this study we sought to explore the clinician and stakeholder perspectives of the experiences of patients and their families qualitatively. The qualitative methods of this study give a voice to those directly affected by the impacts of the pandemic on health systems (i.e., clinicians) and provide detailed insights that add to the existing quantitative literature. Through this qualitative approach we also sought to contextualize the shared experience of patients and families across multiple countries and various global regions. Ultimately, the purpose of this study was to evaluate clinician perspectives on the global impact of the pandemic on children with cancer and their families.

## 2 METHODS

### 2.1 Study design and participant selection

This study is a secondary analysis of previously collected focus group data examining the global impacts of the COVID‐19 pandemic on childhood cancer care. The original study assessed factors of resilient health care during the COVID‐19 pandemic^23^ using rapid turnaround analysis of qualitative data.^24^ For this analysis, we conducted an in‐depth analysis focused on the impacts of the pandemic on children with cancer and their families.

**FIGURE 1:**
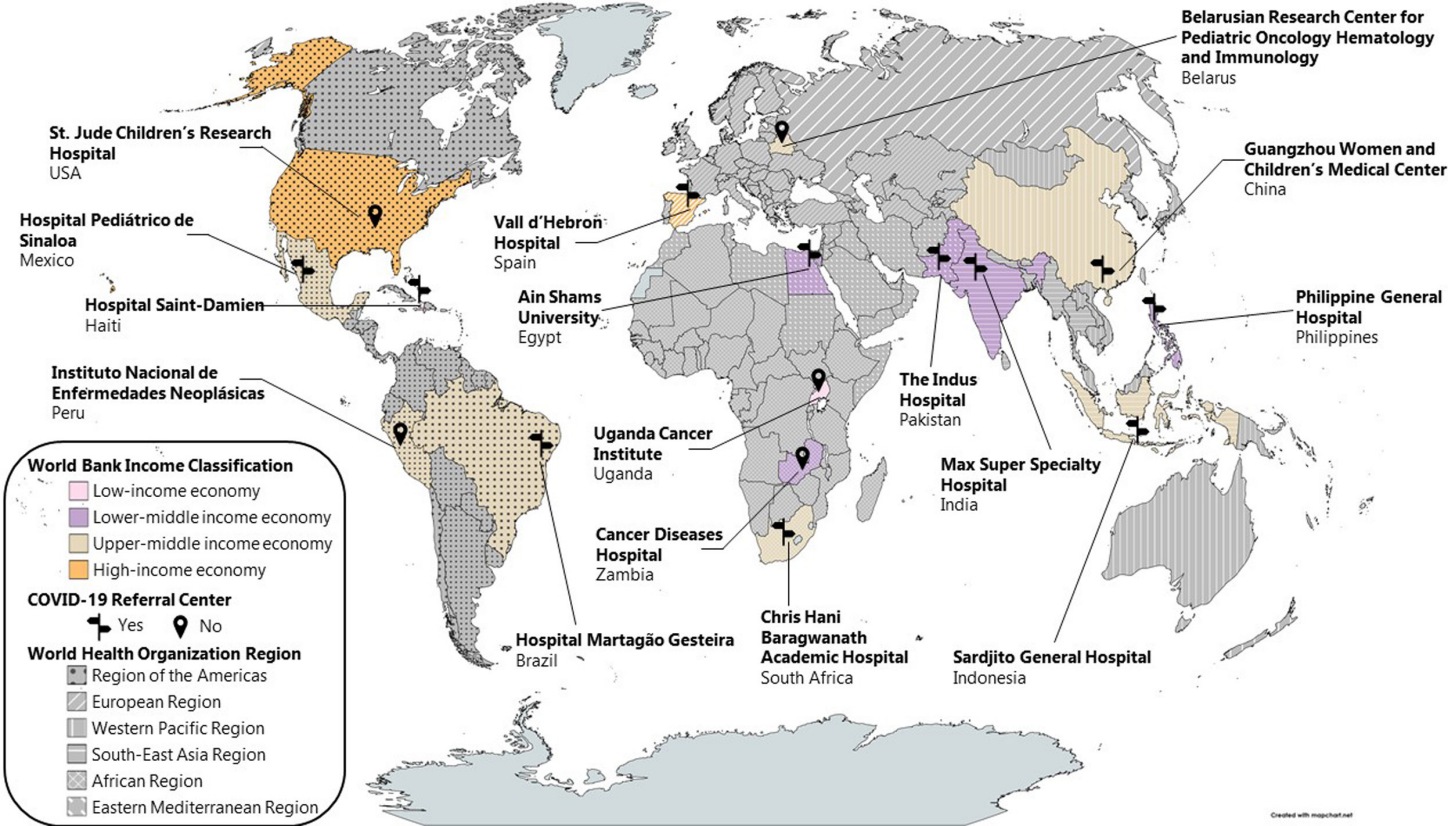
Focus group institutions. This map shows the countries included in the focus groups filled in with one of four colors. The colors indicate which World Bank reported income classification the country belongs to (low‐income, lower‐middle income, upper‐middle income, and high‐income). The flag icon indicates a center identified as a COVID‐19 Referral Center while the pin icon indicates those that were not. All countries are shaded in according to their WHO region.

Details of participant selection have been previously described.^23^, ^25^ Briefly, participants for the qualitative sample were purposively selected^26^ from eligible participants who self‐identified as wanting “to tell us more about your institution's experience during the COVID‐19 pandemic?” in the cross‐sectional survey^24^, ^25^ and to be inclusive of diverse geographical and resource settings and varying income classification levels. The study sample included 16 institutions from 16 countries representing all income classifications and world regions, as defined by the World Bank^27^ and the World Health Organization,^28^ respectively (Figure 1). A total of 19 focus groups were held as three institutions (United States, Philippines, and Spain) chose to hold two focus groups each, with hospital administrators separated from bedside providers according to local principal investigator preference.

### 2.2 Data collection

A semi‐structured focus group guide (Appendix S1) was piloted, iteratively revised, translated into seven languages, back translated into English, and reviewed by bilingual members of the research team to ensure translation accuracy.^29^ Focus groups were held between September 4 and October 27, 2020. Focus groups were led by two facilitators in the local language and were conducted virtually using an online video‐conferencing platform (Zoom or WebEx). Audio recordings of the focus groups were professionally transcribed and translated into English. Focus group transcripts were then de‐identified and reviewed, with the translations validated by bilingual research team members to ensure accuracy.

### 2.3 Data analysis

A codebook (Appendix S1) was developed using novel codes derived inductively by three researchers (MA, EM, PW) through iterative review of six transcripts based on recurring themes identified in the data. Each identified code was defined and refined based on further transcript review until all three members reached consensus. Two researchers independently coded each transcript with one researcher (MA) coding all data and the others each coding 50% of transcripts (EM and PW). After double‐coding, the third researcher acted as an adjudicator to resolve discrepancies and establish consensus. Thematic content analysis identified eight themes related to the impact of the COVID‐19 pandemic on children with cancer and families. Themes were categorized into three domains and compared across Country Income Classifications as defined by the World Bank.^27^ MAXQDA software (VERBI, Berlin, Germany) was used for data management, coding, and analysis. Methods are reported using the Consolidated Criteria for Reporting Qualitative Research (COREQ)^30^ Guidelines to maintain rigor of qualitative reporting.

## 3 RESULTS

Nineteen focus groups were conducted with pediatric oncology providers and hospital stakeholders in 16 countries in 8 languages. The 16 institutions included both publicly and privately funded hospitals. Focus group participant demographics have been previously described^24^ and are included as Appendix S1.

**FIGURE 2:**
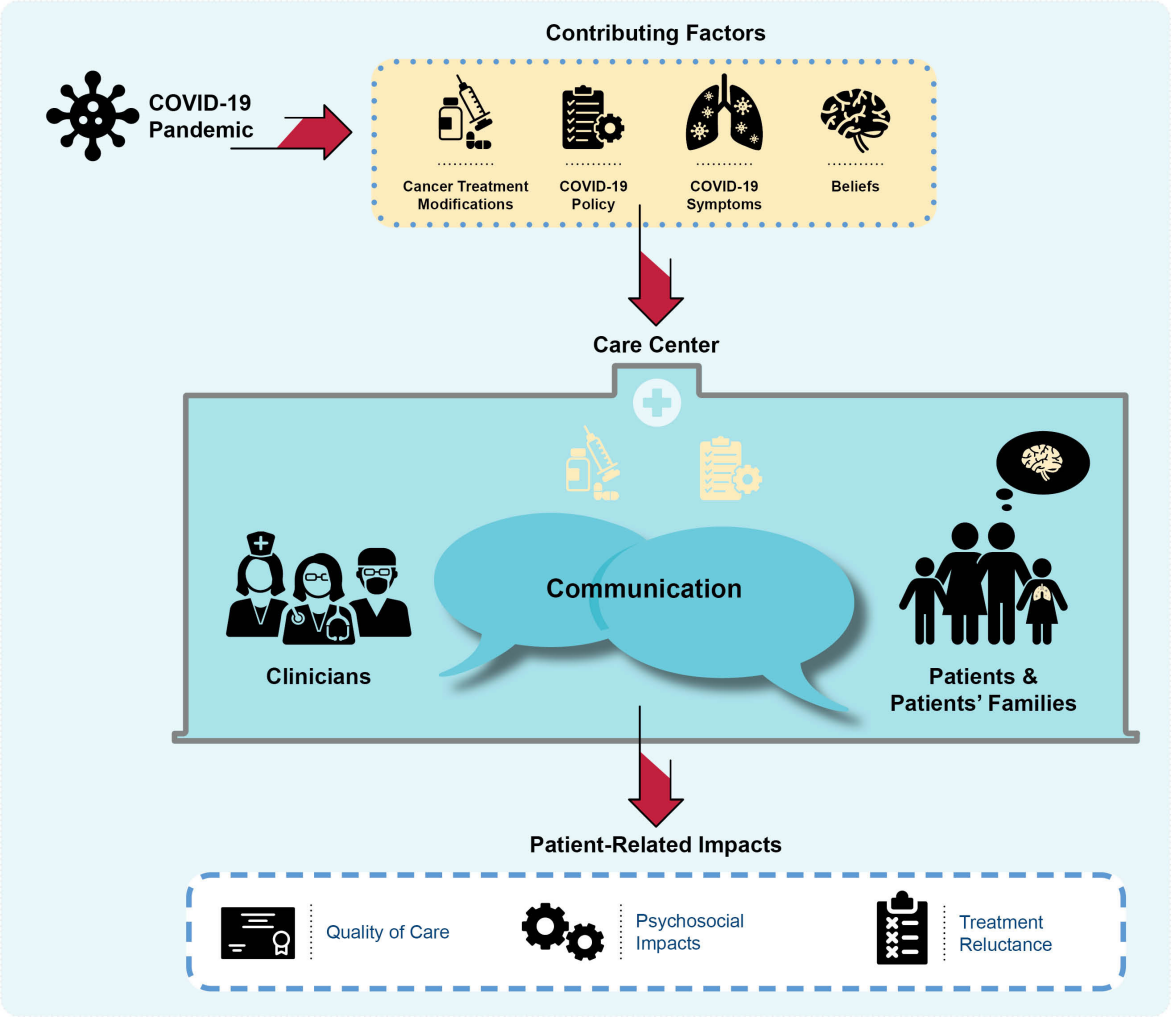
Model describing identified themes. Introduction of the COVID‐19 pandemic led to *Cancer Treatment Modifications* and *COVID‐19 Policy* development, which affected both healthcare providers and pediatric cancer patients and families. Patients and families also experienced *COVID‐19 Symptoms* and personal *Beliefs*, affecting their overall patient care experience during the pandemic. The effect of patient‐related impacts on the patients and families is dependent on the interaction of these components (i.e., contributing factors and impacts) transformed by the central factor: *Communication*. The valence of these impacts depended on the quality and quantity of communication, both between clinicians and with children with cancer and their families.

Participants described eight themes related to the impact of the COVID‐19 pandemic on children with cancer and their families (Table 1). Analysis resulted in categorization of themes into three domains: contributing factors (*COVID‐19 Policies, Cancer Treatment Modifications, COVID‐19 Symptoms, Beliefs*), patient‐related impacts (*Quality of Care, Psychosocial impacts, Treatment Reluctance*), and a central transformer (*Communication*; Figure 2).

**TABLE 1:** Breakdown of eight themes classified into three domains.

Category	Theme
Contributing factors	COVID‐19 Policy
	Cancer treatment modifications
	COVID‐19 Symptoms
	Beliefs
Central transformer	Communication
Patient‐related impacts	Psychosocial impacts
	Quality of care
	Treatment reluctance

Abbreviations: COVID‐19, coronavirus disease 2019.

### 3.1 Contributing factors

Contributing factors resulting from the pandemic impacted patients and families similarly across all income levels (Table 2). *COVID‐19 policies* and *cancer treatment modifications* were implemented by hospital systems as a result of the pandemic. Additionally, patients and families experienced *COVID‐19 symptoms* and had personal *beliefs* regarding the pandemic that impacted their care experience.

**TABLE 2:** Focus group excerpts: contributing factors.

Contributing factor	Excerpt	Importance/Implications
Factors brought into the hospital system as a result of the COVID‐19 pandemic		
COVID‐19 Policies	“I know the visitation policy changed quite a bit in the ICU particularly around kids at the end stage of life…hospitals all over the country changed that policy. And that was developed through the incident command center…But that was probably the most dramatic and difficult policy for the ICU to adopt. And I really hope that goes away because it's been bad.” (USA)	Mandates at departmental level in response to the COVID‐19 pandemic impacting patients and their families
	“Also, among the measures taken in the COVID crisis, the hospital provided masks not only for patients, but for their families as well. Beside masks in the inpatient and outpatient clinic, so that we do not deal with any client, unless wearing a mask, as well as all of escorts. It was a routine followed by patients and their families also to wear masks…Before COVID‐19, a patient was allowed to be escorted with more than one. Now, only one family escort is allowed. Beside restricting visits in the internal section.” (Egypt)	Mandates at hospital level in response to the COVID‐19 pandemic that impacted patients and families
	“What changed a little bit, is the hospitalization algorithm [organizational structure]. Patients, mostly every one, before they are examined, before hospitalization, they are put in, what is called, and observation period, in which we try to isolate them in a separate room, before their COVID tests come back. If their test results come back negative, then we admit them to general hospital ward, meaning a two, three, or four‐person room” (Belarus)	Mandates at the hospital level related to the COVID‐19 pandemic
	“The government had issued even a statutory instrument requiring masking in public places. And that continues up to now.” (Zambia)	Mandates at governmental level in response to COVID‐19
Cancer treatment modifications	“[There] have been delays in certain of our services. For example, surgery…because…they had scaled down all non‐emergency surgeries” (Uganda)	Delays in care due to COVID‐19 pandemic
	“…we canceled all surveillance consultations so that they would travel less, so as not to put them at risk…” (Mexico).	Changes in schedule as a result of the pandemic
	“And in fact, we have been offering world class surgery to all patients from all parts of North India right from Patna which is in the northeastern part of India or in Eastern state so Punjab to Uttar Pradesh. So, far of places children can now come and get this surgery done and go back to the place where they were getting their chemotherapy. And that has kind of continued even in the Covid times…” (India)	Sustained care during the COVID‐19 pandemic
	“And, of course we changed course, a little bit, on our chemotherapy treatment procedures, may be you have heard or know that the recommendation, against the use methotrexate in high doses, if the coronavirus is present, the complete stoppage of chemotherapy, if diagnosed with the virus, or the postponing of the dates of chemotherapy treatments. There are of course international recommendations, which we try to follow, so that means, that the way we go about chemotherapy treatment, concerning our/those patients, has changed.” (Belarus)	Coronavirus positivity impacting cancer treatment
Factors experienced by patients and families during the COVID‐19 Pandemic		
COVID‐19 symptoms	“Before, it was a problem when an oncology patient would come to the emergency room because if he had respiratory symptoms, they would send him right away to the COVID area. And we would argue why, it could be a neutropenic fever, a simple respiratory infection. There was that stress…and right now that is already more protocolized…” (Mexico)	Addresses challenge distinguishing COVID‐19 symptoms from side effects and/or symptoms of other illnesses
	“Especially since we had seen here in Haiti children with positive Covid who are completely asymptomatic, it is from there that we were afraid especially for the children who come to the [oncology] service, we do Covid test there is yes or no a type of symptomatology? To know if we do the isolation” (Haiti)	Effects of asymptomatic COVID‐19 on patient workflow
Beliefs	“So we really restructured from a logistical point of view by disinfecting some rooms and buildings to have a building separate from the big hospital building to receive children who would be positive for Covid, that's how we have a wing in the hospital that is really separate from the big hospital that we had called IRA department to avoid stigmatization. Because at the beginning in Haiti we started to stigmatize people who were declared positive for COVID‐19, so we did not want the public to stigmatize the place, so we called it the IRA department.” (Haiti)	Stigmatizations around COVID‐19 influenced general community's perception of hospitals
	“So what the parents were doing…is a denial of the disease, there is a whole stigma of the disease so some children were either leaving outright, or they were hiding them the parents were hiding the symptoms” (Haiti)	Stigma around COVID‐19 impacted parent's response to their children's symptoms
	“…It's not like other rooms at the hospital where they only have 2 patients. We have an area with 12 patients, another with 10 patients, another with 11 patients, and the parents…their level of education is low and they do not comply with the rules, so the technical nurses must be like babysitters, repeating constantly, Sir, please, keep your distance. Sir please, stay on your seat. And we even had some cases where they disrespected the staff…This is why we had to stop receiving patients in order to disinfect all the areas.” (Peru)	Patient and family education levels impact on response and compliance to COVID rules

Abbreviations: COVID, coronavirus disease; COVID‐19, coronavirus disease 2019; ICU, intensive care unit; IRA, acute respiratory infection.

*COVID‐19 related policies* mandates affecting patients and families were determined by departments, hospitals, and governments. Hospital policies included lockdowns, scaling down of hospital services, COVID‐19 testing, masking, sanitation, and other protocols: “…there are new rules in terms of social distancing and…in terms of wearing a mask in the hospital all the time…also the hospital has become…stricter in terms [of] the number [of visitors] allowed in an area. So, sometimes it's difficult especially for palliative patients…to say that you can only really have one [visitor]” (Philippines).

*Cancer treatment modifications*—including delay of care, schedule changes, inability to transfer to other hospitals, access to medicines—were commonly discussed. Delay of care included delays in diagnostic imaging and surgical procedures. Clinicians described difficulty outsourcing specific treatments because collaborating hospitals closed or had limited services. Scheduling changes ranged from shortening visits for chemotherapy administration, “all those children who are planned for chemotherapy…for some of them we had to shorten these days in the hospital…[so] children will…spend less time in the hospital to reduce their exposure to Covid” (India) to canceling consultations. Participants also described parents requesting modifications to their child's treatment to limit time spent at the hospital: “Some of the parents…said okay please give us eight or nine do not give us more cycles because we do not want to come to hospital very often because they were scared their child would get Covid” (India). Other commonly discussed impacts on cancer treatment were blood product shortages and decreased access to cancer medications: “…a lot of our chemotherapeutic agents are imported from overseas and so…we have had drug shortages…” (South Africa).

Despite treatment modifications, participants described their ability to provide care throughout the pandemic: “…during the whole period, our pediatric oncology services never stopped for a single day” (Pakistan). Participants emphasized the effort and teamwork required to sustain cancer treatment: “…to manage to keep the most essential activities…it was a hard effort from everybody, and the fact that we could keep all the pediatric hemato‐oncology activity without suspending any treatment…it was a tremendous effort” (Spain).

Although some children with cancer and their families with COVID‐19 were asymptomatic, many directly experienced *COVID‐19 symptoms*, and at times it was challenging to distinguish COVID symptoms from those of other illnesses or side effects of the child's cancer therapy. When patients came into the hospitals with asymptomatic COVID‐19, it created additional stress: “…in children there are not really typical signs of Covid, so any child in the hospital can be a case of Covid and there's always a little bit of stress about it” (Haiti). Clinicians also emphasized the impact COVID‐19 positivity had on patients' cancer treatment, including inability to receive chemotherapy: “…second aspect was due to COVID positivity, we were unable to deliver the treatment to our patients, chemotherapy” (Pakistan).

In addition to symptoms, participants discussed patient and family *beliefs* that impacted the child's care. Participants emphasized stigmatization surrounding COVID‐19 and how it led parents to hide their child's symptoms (e.g., a cough) from healthcare providers. General community stigma about COVID‐19 also impacted perceptions of hospitals: “…there is still a lasting stigma about hospital becoming ‘the nest of COVID‐19’. Therefore, people do not want to visit hospitals…” (Indonesia).

Clinicians perceived cultural context and education affecting the *beliefs* families held about COVID‐19 and their response to implemented policies: “…[it] was a bit difficult to manage…the social distancing between parents. It's a cultural thing with us that when we have someone who is sick, we sympathize a lot…And even when you attract attention and try to make them realize that this was not possible in times of pandemic, but it was difficult because it was a habit that had become part of their culture and their way of doing things” (Haiti).

### 3.2 Patient‐related impacts

Ultimately, participants discussed three pandemic‐related impacts that affected patients and families similarly across the world: *quality of care, psychosocial impacts, and treatment reluctance* (Table 3).

**TABLE 3:** Focus group excerpts: patient‐related impacts.

Patient‐related impact	Excerpt	Importance/Implications
Quality of care	“We want to keep some of these measures in place because we have noticed there has been an opportunity also to improve the quality of care that we are providing our patients. So, in some respects, the changes have been positive on their patients. We see, well, when COVID started we are seeing fewer patients and we are spending more time per patient. Now the patient numbers have gone back up, but we are still trying to manage with a given restrictions…” (Uganda)	Describing increased quality of care: decreased patient load allowed for increased time spent with patient
	“…in terms of the palliative care…that we provide for kids who aren't supportive. And I think that service was affected very severely by the pandemic.” (Pakistan)	Negative impacts of the pandemic on palliative care services
	“…At that time, because there was a misunderstanding, especially in nursing, sometimes we missed out on the critical care of our patients who were admitted into the COVID PICU, so we ended up in a situation that we did not want, i.e., death…there is a rule among nurses that we can only go in [during specific hours]. So, in PICU COVID, there is a nurse room and a patient ward and there are two layers of doors. We can only go in with PPE level 3 during specific hours; for example, 6 AM, 12 PM, 6 PM, 10 PM. If there is an emergency situation outside of these hours, we miss treating these patients and our patients ended up in worse condition.” (Indonesia)	Degradation of care due to changes because of COVID
	“I think one of the things that helped our patients is a program called Aprendo Contigo [I learn with you], it was about hospital schooling. It helped children to face all this process and the illness and its treatment. Unfortunately, due to the prevention of exposure, it's been canceled. Another thing within the support and the treatment of these patients, it's the matter of blood transfusion. The percentage of people donating dropped a lot and there was a huge campaign to encourage people to donate. We have one of most common pathologies which is leukemia and it has a huge demand for transfusions…” (Peru)	Reduction in supportive services (hospital schooling) offered as a result of the pandemic in addition to blood supply shortages.
Psychosocial	“Since the quarantine start, we do osteosarcoma mental health every month. Yes. We do communicate with our patient how they feel regarding this pandemic. How are they doing. Second…how will they adopt to that? We're helping them to adopt in this pandemic.” (Philippines)	Provision of psychosocial support
	“Also, we used to do a lot of group therapies for our patients. So, patients would come in, they could talk to each other, and that was also hampered…And we were not allowing the children to access…the play area. So, parent mentoring was also stopped. We could not continue with our support groups that we did for our patients. So, I think those are the ways that were negatively affected because of the pandemic.” (Pakistan)	Decreased psychosocial support for patients and parents
	“So one of the big issues that patients have had this actually just get into the hospital physically because there's no public transport, there's a limitation on movement, so people have this general sense that they are not going to be allowed to go out, if they do go out they are going to encounter police checkpoints it will just be too much of a hassle.” (Philippines)	Issues with transportation as a result of the pandemic
	“We also had a lot of difficulties in the ICU with the parents…And they were really scared, they showed a lot of difficulty to understand, to understand that they could not leave…For example, the issue of clothes. As we receive many families from the countryside, they come with suitcases, they come with the clothes from home and they have no way to take them to another place to wash…So it was a period, and it is still a very complicated period in relation to this. We really had a lot of support from the social service to help with these issues.” (Brazil)	Lack of social services support for patient families
	“…The parents do not have the money to bring them back to the hospital and then schools were closed. So a lot of the youngest siblings had to be left alone at home. So those were some of the challenges in terms of the patients getting treatment.” (South Africa)	Social factors impacted by the pandemic affecting patients' ability to receive treatment
	“…Indeed, the integral part has been reduced, including the psychological part and support. The only psychiatrist coming right now…psychology residents are not coming to work. We only have 2 psychologists for the entire hospital, and they must deal with everything. From the integral point of view, that has been affected as well. The emotional part and the support our patients had…” (Peru)	Decreased mental health support available
Treatment reluctance	“And many patients got LAMA, Left Against Medical Advice, they abandoned. And they took the child home that we will not get tests being done. And even few patients were admitted. And when they got COVID positive during stay, and we were shifting to the COVID unit and they got abandoned and they refused for treatment. So, we had lot of abandonment during this pandemic due to COVID positivity…” (Pakistan)	Patient left against medical advice (i.e., changes in patient cancer treatment) as a result of COVID‐19
	“Of course, unfortunately we lost some children to retinoblastoma who would not comebacks and there some who have not been followed up properly because of the transport challenges that occurred. So, it's been an opportunity at the same time. The challenges are there but we have learned some things that we maximize on the time that we get with a patient would work so hard to make sure the investigations are hurried and the see that we get to the point of surgery in the next phase.” (Uganda)	Patient was not able to return to the hospital due to impacts of the COVID‐19 pandemic on transportation

Abbreviations: COVID, coronavirus disease; ICU, intensive care unit; PICU, pediatric intensive care unit; PPE, personal protective equipment.

Some participants described an increased *quality of cancer care* due to policy implementation, which decreased patient load and thus increased clinician time per patient. However, staff shortages and longer shifts resulted in perceived degradation in care quality: “…one important thing we experienced during this time was the overload of work. Most…[of] the nursing staff have had COVID and during our recovery time no additional staff was sent to our areas. So, they would assign us 5 patients instead of 3, and that affected the quality of patient care…” (Mexico).

Focus group participants described maintaining a high level of medical care but providing less holistic care: “Medically, yes we do great…But overall holistic approach to the patient…I think it has changed dramatically” (USA). Clinicians detailed how some care was managed through home visits, but certain supportive care services, such as physical therapy, were not available: “We have quite a few teenagers who have been receiving practically exclusive home care and the only thing that would have given them an extra quality…would have been to do some more physical therapy, which has not been possible due to the movement restrictions” (Spain). Additionally, some participants discussed the negative impact the pandemic had on palliative services while others described these services being transitioned to home care: “In oncology we have a good number of patients in palliative care, so we had restructured for them as well and we based ourselves on home care” (Haiti).

Furthermore, participants mentioned the *psychosocial impacts* that policies and the loss of support services had on patients and families: “Because of COVID, our volunteer team cannot come to the ward community onsite. Considering the boring life of long‐term inpatient children, volunteers bring lots of toys and masks to our kids…our medical staff may engage kids with toys in some interactive games. Indeed, we cannot do it as well organized as our volunteer teams…” (China). Patients and families also experienced issues with transportation. Some participants described how hospitals addressed these challenges by working with NGOs to ensure children had transportation to the hospitals: “We have so many patients from outside of Delhi…So, without having arranged that transport they wouldn't have reached the institute's they were intended to, so that was a real support” (India).

Clinicians also noted an increase in *treatment reluctance*. Participants described families stopping or pausing treatment due to fears of contracting COVID‐19 and reluctance to take a COVID‐19 test or stay at the hospital: “We also had patients who feared to come even when there was transport offered…There are those who are really so scared of the disease and they are telling us Covid is in Kampala…They wouldn't come back because of that trauma they had about Covid” (Uganda).

### 3.3 Central transformer

Communication transformed how contributing factors affected patient‐related impacts. Communication was mentioned more in centers located in higher‐income countries compared to centers in low‐income settings (Table 4).

**TABLE 4:** Focus group excerpts: central transformer.

Central transformer	Excerpt	Importance/Implications
Communication between clinicians	“But in Karachi Indus provide support to many institutes in Karachi. Many institutes I must say and provide them free of cost PCR testing for their patients who need to be admitted or who need to [undergo] any prevention or any procedure.” (Pakistan)	Communication between facilities to ensure all patients were tested so they could receive care
	“One of the best things that has come out of COVID is Zoom. The ability, the ability to actually share knowledge or have those to my board either at the comfort of your home or in your car, and you are actually able to solve a problem or to actually reach a consensus on patient management without physically being in the same room…” (Uganda)	Virtual communication between clinicians allowed for continuity of care
	“Regarding the collaboration with other centers, we usually collaborate with other hospitals in Catalonia and the rest of Spain. This has been so difficult for us to do our best to ensure that patients travel as little as possible during the peak season. We have many patients who come to be treated in clinical trials, for example who come from 600 km or 1000 km, very distant places, and it has also been an effort to continue working with these teams” (Spain)	Communication between providers to continue collaborations while also wanting to minimize patient exposure
Clinician‐patient interactions	“…I had a nurse tell me that a dad got down on his knees and begged to not abide by the single caregiver rule at which we were only allowing one caregiver to stay. And I think that was one of the hardest things that the nurses had to do was to make families leave because that was just so counterintuitive because what [institution] nurses have been taught their whole lives is you…do whatever it takes to take care of the patient and family and make this the best experience possible.” (USA)	COVID‐19 policy driving change in clinician‐patient/family relationship
	“…Not only do I hate not being able to…use my face to communicate with a patient or a caregiver…[the PPE] makes me feel more distant than even the six feet between us. And it makes it harder…” (USA)	COVID‐19 policy limiting clinician‐patient family connection
	“…In terms of patient care, one thing that was decided upon…was to as much as possible, reduced the number of post treatment follow ups. And so we, the nursing team through colleague [has] been communicating the patients scheduled for review, all those that would call in to find out if they were to come for reviewing to, reviews have had to be rescheduled appropriately. All those that have to come on an outpatient basis but on active therapy, we have continued offering the service uninterrupted…” (Zambia)	Consistent communication between clinicians regarding limiting visits due to various COVID related factors allowed for organized communication with patients and continued care
	“That means, that planned surgeries and surgical procedures were postponed, if the patient was in contact with the virus. Information like this was given to them on the phone. The patient was informed.” (Belarus)	COVID‐19 infection and policies limited available care services and virtual communication allowed for clinicians to still communicate with patients and families
	“…Everyone was scared all the time. The nursing staff usually guides the parents and they call the parents on arrival at the anti‐bedroom and guide them since the use of clothes, hand sanitizing, not using the cell phone, these things. And we had a reinforcement with the social service staff as well. They would go up just after the admission, besides reinforcing all this, they would also give more assistance because as they are isolated from the world…So there was a bigger job I think of social service too.” (Brazil)	COVID‐19 regulations impacted (1) what was communicated and (2) the amount/importance of the social services team's interaction with patients and families
Virtual communication	“Also I noticed that a few kids who could have been diagnosed maybe, you know, 15 days or a month prior to the initial diagnosis came a little later…because the parents were scared of actually going to the doctor or our hospital and we know physical visit rather many of these kids were doing telephonic visits in the initial time when…lockdown started in our country. So, the diagnosis was also delayed.” (India)	Virtual communication allowed for visits to occur but interaction and capabilities were limited
	“…we had trained the parents at each visit, we had a WhatsApp group, we had phones available for them, they had to call before arriving at the hospital if it's necessary we refer them, if it's necessary to come then they come, if it's the field agent who should come then he comes” (Haiti)	Virtual communication utilization in low‐income country
	“…So, the truth is that at first it happened that there was no adherence to treatment in the sense that the parents did not want to go to the emergency room. For example, they know of the importance of the Golden Hour, of the symptoms, of going early, but they thought that if they came to the emergency room, they were going to get COVID or they were going to get more serious. So, what I saw is that the demand was for the doctors to attend them by phone. Hello, he has this … no, you have to come to the emergency room…” (Mexico)	Virtual communication facilitated clinician‐patient and family communication during the pandemic

Abbreviations: COVID, coronavirus disease; km, kilometer; PCR, polymerase chain reaction; USA, United States of America.

*Communication between clinicians* mitigated the effect the pandemic had on patient‐related impacts. Participants described how patients and families were reluctant to go to the hospital because of COVID, and how clinicians communicated to ensure care delivery: “If it [isn't] life threatening people don't want to go to the hospital or there's also a general sense that the hospital is a dangerous place to be in so…[we] work with other teams in other places to try to capture those patients and have them have regular follow ups with these other teams” (Philippines). Patients' and families' *beliefs* about testing often reduced their desire to visit the hospital, but when clinicians listened and took time to communicate with them, their understanding improved: “We always explain to the patient, because they are pediatric…[the test] will hurt, it will bother a little but it is necessary…I always tell them…even if he doesn't let me do it, you will see that he will understand…” (Mexico).

Additionally, consistent *communication between clinicians* about policies positively impacted *clinician‐patient interactions*: “When there is a fluid communication channel between the entire team, they leave with the same message. And when we all go with one message; this gives a lot of peace to the families and also to the professional himself because you know that we are all on the same line. And in this regard, families, like us, that have to adapt and tolerate uncertainty…are given a clear message despite being a message that is not to their liking, such as reducing visits, like having only one caregiver, but it gives security, it gives a framework of mobility” (Spain). However, some COVID‐19 policies limited *clinician‐patient family interactions*, negatively impacting care: “…the parents couldn't visit. So it was very difficult to communicate a new diagnosis or to get consent for certain investigative procedures, if you cannot sit in the same room and discuss that with a parent” (South Africa).

*Virtual communication* positively impacted *quality of care* and mitigated the effects of *policies* on the flow of patients at the hospital. However, participants also described how *policies* impacted families' abilities to be with patients and how specific conversations were difficult to have virtually: “…the hardest thing for me is…having to have these discussions with families about bad news. And it's so difficult…to have that discussion with one parent on FaceTime and the other parent in the room…or when a patient's crashing and I'm trying to figure out because they have relapsed disease that has no treatment. Do they really want me to intubate them or not” (USA).

Lastly, participants mentioned how *virtual communication* provided patients and families an outlet to discuss their experiences during the pandemic: “…one of the first things that happened was a 24‐h hotline was set up for the patients and families to call, and it's still up and running today. She gets a lot of calls…But honestly, the majority are somebody who just wants to talk…and they just need to some way to express their frustration, and they understand why the rules are in place but it's hard…” (USA).

## 4 DISCUSSION

The COVID‐19 pandemic impacted healthcare systems and altered pediatric oncology care worldwide.^4^, ^21^ This study demonstrates how clinicians perceived communication practices transforming the effect of the COVID‐19 pandemic on patient‐related impacts. The use of telehealth services allowed for continued communication between patients and clinicians when in‐person consultations were canceled or postponed due to implemented policies.^11^, ^31^

Additionally, communication between clinicians within the same institutions and across partner institutions contributed to positive interactions with patients and families. This ultimately improved patient‐related impacts, including psychosocial outcomes and treatment reluctance. Previous studies reported increased anxiety and post‐trauma symptoms among parents of children with cancer,^18^, ^19^ which were exacerbated by the loss of social support due to pandemic policies and constant fear of COVID‐19 infection. Furthermore, the importance of developing communication policies structured around clinician communication has been demonstrated to protect healthcare providers and thus mitigate impacts of the pandemic,^32^ ultimately improving the quality of care provided. Healthcare institutions should consider implementing policies focusing on how new or changes to existing hospital policies are communicated to patients and families during pandemics in order to mitigate the impacts felt. Additionally, policies implementing guidelines for clinician communication and addressing psychosocial support for clinicians should be explored by healthcare organizations. Our results reinforce the importance of teamwork and communication to improve the overall care experience for children with cancer and their families during a pandemic.

Communication is a practicable intervention clinicians can utilize in everyday care that can have a strong positive impact on patients and families. Communication training should be prioritized for clinicians as it can be highly impactful on the quality of care^33^ and can increase utilization of communication innovations (e.g., chat groups and other virtual forms) during times of crisis. These findings can guide hospital stakeholders and clinicians to implement interventions that improve both interdisciplinary team communication and patient‐centered care.

Clinicians also perceived education level and cultural context as impacting the beliefs held by patients and families and their responses to the COVID‐19 pandemic and its effects. Previous studies investigated the role of education level in knowledge, attitudes, and practices related to COVID‐19 and found a higher education level was associated with increased knowledge of COVID‐19 and higher attitude scores, or a more positive attitude, as compared to those with a lower educational level.^34^, ^35^ The perceptions of the participants in our focus groups support the previous findings' conclusions that educational interventions are needed and should be sensitized to factors like the population's education level.^34^, ^35^

This global analysis shows how the COVID‐19 pandemic similarly impacted the care of children with cancer in countries across World Bank income classifications,^27^ adding to previous literature describing regional, country, and single institution studies.^4^, ^13^, ^14^, ^36^ The three domains—contributing factors, patient‐related impacts, central transformer—were consistently identified across all country income classifications, with the central transformer, *communication*, mentioned less often by participants in low‐income countries. This difference could be due to a bigger focus on allocation and mobilization of physical resources in resource‐limited settings^7^, ^37^ with less exploration of the utility of communication efforts. It is also possible the pandemic more greatly impacted patient care and communication in these settings, potentially increasing existing disparities in childhood cancer care.^38^ These differences suggest the need for more ongoing support for low‐income countries and the consideration of local context specific challenges (e.g., technology accessibility) in developing communication policies to address current gaps in accessibility as well as help governmental response during future pandemics^39^ and ultimately mitigate the impact on patients and families.

Using qualitative methods allowed us to detail clinician and stakeholder perspectives on the impact of the pandemic on children with cancer and their families in‐depth and provided insights beyond what was previously reported quantitatively, including evincing the important role of communication in transforming those effects and the intricacies of COVID‐19 related policies and their impacts. Including a diverse, multidisciplinary group of clinicians and hospital stakeholders allowed us to encapsulate the shared experiences of participants across different settings that can be used to inform interventions and policies on different levels in response to future global health crises. The multilingual nature of this study was a strength as it allowed global participation from diverse clinicians. This study, however, has several limitations. All analysis was conducted in English. To minimize misinterpretation, a bilingual, native‐speaking member of the study team reviewed transcripts to verify translation accuracy. We report the effects of the pandemic on children with cancer and their families as perceived by clinicians; patients and families were not included in this study and the initial study guide wasn't framed around this question. Rather, the need for this secondary analysis became evident due to data arising through spontaneous comments from participants about their perceived effects of the pandemic on patients and families and the impact of communication. Although these findings provide a starting point regarding potential impact on families, future research should address the effects of the pandemic from the patient and family/caregiver perspectives. Additionally, future research should continue to investigate how alternative factors, such as hospital resources and policies, impacted patient‐related outcomes and how they can inform interventions and policies.

## 5 CONCLUSION

This global assessment highlights clinician's and stakeholders' perceptions of how communication transformed the impact of the COVID‐19 pandemic on children with cancer. This study further emphasizes the importance of communication in patient‐centered care and supports the development of low‐cost communication interventions throughout the healthcare system to improve childhood cancer care globally.

## AUTHOR CONTRIBUTIONS

**Gia Ferrara:** Formal analysis (lead); writing – original draft (lead). **Molly Aguina:** Formal analysis (equal); writing – review and editing (equal). **Emily Mirochnick:** Data curation (equal); writing – review and editing (equal). **Parima Wiphatphumiprates:** Data curation (equal); writing – review and editing (equal). **Daniel C. Moreira:** Conceptualization (equal); investigation (equal); writing – review and editing (equal). **Elizabeth Rose Sniderman:** Investigation (equal); writing – review and editing (equal). **Cesar A. Villegas:** Investigation (equal); writing – review and editing (equal). **Erica Carmen Kaye:** Investigation (equal); writing – review and editing (equal). **Iman Ragab:** Investigation (equal); writing – review and editing (equal). **Biemba Maliti:** Investigation (equal); writing – review and editing (equal). **Gita Naidu:** Investigation (equal); writing – review and editing (equal). **Pascale Y. Gassant:** Investigation (equal); writing – review and editing (equal). **Daniela Arce:** Investigation (equal); writing – review and editing (equal). **Ramandeep Singh Arora:** Investigation (equal); writing – review and editing (equal). **Ana Patricia Alcasabas:** Investigation (equal); writing – review and editing (equal). **Muhammad Rafie Raza:** Investigation (equal); writing – review and editing (equal). **Pablo Velasco:** Investigation (equal); writing – review and editing (equal). **Joyce B Kambugu:** Investigation (equal); writing – review and editing (equal). **Anna Vinitsky:** Investigation (equal); writing – review and editing (equal). **Carlos Rodriguez Galindo:** Resources (equal); writing – review and editing (equal). **Asya Agulnik:** Conceptualization (equal); investigation (equal); methodology (equal); writing – review and editing (equal). **Dylan E. Graetz:** Conceptualization (equal); formal analysis (supporting); funding acquisition (equal); investigation (equal); methodology (equal); writing – review and editing (lead).

## FUNDING INFORMATION

Funding support to St. Jude Children's Research Hospital provided by the Cancer Center Support (CORE) grant (CA21765) and the American Lebanese‐Syrian Associated Charities (ALSAC).

## CONFLICT OF INTEREST STATEMENT

The authors declare no competing interests.

## ETHICS STATEMENT

The institutional review board (IRB) at St. Jude Children's Research Hospital (SJCRH) reviewed and approved the study with SJCRH as the coordinating center. Additional review and approval were obtained locally as required. All participants provided verbal consent for participation and recording.

## Supporting information


Appendix S1

## Data Availability

The data that support the findings of this study are available from the corresponding author upon reasonable request.

## APPENDIX A

### A.1

COVIMPACT Study Group Collaborators: Aliaksandra Laptsevich MD, Belarusian Research Center for Pediatric Oncology Hematology and Immunology, Minsk, Belarus; Haiying Huang BSN, Guangzhou Women and Children's Medical Center, Guangzhou, China; Luciana Nunes Silva MD, Hospital Martagão Gesteira, Salvador, Brazil; Jacqueline Montoya Vasquez MD, Instituto Nacional de Enfermedades Neoplásicas, Lima, Peru; Desy Rusmawatiningtyas MD, Sardjito General Hospital, Yogyakarta, Indonesia; Nancy S. Bolous MD, Cyrine E. Haidar PharmD, Laure Bihannic PhD, Diana Sa da Bandeira PhD, Jade Xiaoqing Wang PhD, Dongfang Li PhD, Flavia Graca PhD, Aksana Vasilyeva PhD, St. Jude Children's Research Hospital, Memphis, Tennessee, USA; Harry Lesmana MD, Cleveland Clinic, Cleveland, Ohio, USA.
