# Supplementary material for: Communication transforms the impact of the COVID‐19 pandemic on children with cancer and their families
Source: Cancer Med. 2023 Apr 20;12(11):12813–26. doi: 10.1002/cam4.5950 (PMC10278468; doi:10.1002/cam4.5950)
Supplement: Supplementary file 1 — Appendix S1 [file CAM4-12-12813-s001.docx]

**Supplementary Appendix**

| **Section** | **Page** |
| --- | --- |
| Supplement 1: COVIMPACT Focus Group Question Guide | 2 |
| Supplement 2: Codebook | 4 |
| Supplement Table 3: Focus Group Participants | 5 |

Supplement 1. COVIMPACT Focus Group Guide

1. Overall, what do you think about your hospital’s response to the COVID-19 pandemic?
2. How has COVID-19 affected or changed the way you work together as an interdisciplinary team?
   1. What creative changes has your team made to enable teamwork under this new stressor?
   2. How has your team worked to support each other?
   3. Has the way you’ve worked as a team changed over the course of the pandemic?

1. How has your team collaborated with other centers, either locally, regionally or around the world?
   1. What types of external support have you received?
   2. What types of external support have you provided?
2. Are there new hospital or unit/ward policies?
   1. If so, what has been the process to develop these policies, as far as you know?
      1. How has your team been hearing about policy changes at your hospital?
      2. How have these policies been used by your team?
   2. If no policies or guidance have been developed, what would be helpful for your team?
3. Has your hospital or unit/ward instituted any new protocols, algorithms or checklists to implement these policies?
   1. Can you tell us about how you have used these?
   2. Have you found these protocols/checklists helpful? Harmful?
4. How has COVID-19 affected or changed the way you work with families/patients?
   1. What creative changes has your team made to enable continued patient care under this new stressor?
   2. How has your team communicated policy changes at your hospital to families?
      1. Are there examples of when this was done well?
5. Of all the changes that have been made, are there any that you think will persist after this pandemic is over? Has anything changed care for the better?
6. Suppose that you were in charge and could make one change that could help your team during this time, what would you do? ***(Have every participant answer this question).***
7. We have taken this opportunity to discuss how your team and institution have responded to the pandemic thus far. Before we wrap up, is there anything else we haven’t covered that you want to mention about how your institution and team have adapted during the pandemic?

Supplement 2. Codebook

| Category | Code | Definition |
| --- | --- | --- |
| Healthcare Treatment | Covid policy | The reference of non-treatment related COVID-19 regulations or guidelines made by hospitals or governmental organizations in response to the COVID-19 pandemic that impacts patients and families |
|  | Cancer treatment | References to variations or lack of variations in cancer treatment (Chemotherapy, surgery, radiation therapy, provision of treatment inpatient vs. outpatient) as a result of COVID-19 on the hospital's part. May refer to specific patients or groups/populations of patients. Also includes impacts felt as a result of these changes. |
|  | Changes in quality | Specific references or descriptions by providers to the improvement or degradation in quality of care; good/bad |
|  | Supportive care | Changes to the patient's hospital care unrelated to curative treatment or psychosocial support |
|  | Symptoms | The reference or the effect of the direct physical symptoms of COVID-19 or the lack of this presentation directly on the patient |
| Social Impact | Psychosocial | References to the psychosocial wellbeing of patients and families including provision of mental health support (or lack thereof) in the form of family members or professionals, as well as stressors included but not limited to finance, housing for families, transportation, or other social resources as it impacts families. |
|  | Beliefs | The impact of the general community's or the patient/family's cultural understandings including religious beliefs or family's level of education as it impacts the patient/family's perception or response to Covid |
|  | Treatment reluctance | Patient or family-members-driven changes in patient's cancer treatment or reservations about seeking treatments as a result of Covid or as a result of fears relating to Covid |
| Interactions | Provider/patient interaction | Changes in the provider/patient and/or family relationship stemming as a result of the effect of COVID-19 regulations as well as the communication between the two; teaching by the provider to the family and indirect communication such as posters; includes provider fear that impacts patient/family |
|  | Communication between providers | Talking between hospital staff within a singular hospital or between hospital staff or between facilities as it impacts the patient/family. |
|  | Virtual communication | Telemedicine or virtual communication SHOULD ALWAYS BE DOUBLE CODED WITH "Communication between providers" or "provider/patient interaction" |
| Impact | Other | Miscellaneous references that influence the impact of COVID-19 on patients/families |

Supplemental Table 3: Focus group participants

| Institution Name  (Total Participants)  Country | Focus Group Language | Physicians (n) | Nurses (n) | Other Roles (n) |
| --- | --- | --- | --- | --- |
| **Ain Shams University** (8)  Egypt | Arabic | Pediatric oncologist (6) | Nurse (1)  Nurse Supervisor (1) |  |
| **Belarussian Research Center for Pediatric Oncology Hematology and Immunology** (4)  Belarus | Russian | Infection control physician (1) | Head nurse (1) | Data manager (1) Head of infection control (1) |
| **Cancer Diseases Hospital** (10)  Zambia | English | Pediatric oncologist (1) | General nurse (2) Pediatric nurse (5) Palliative care nurse (1) | Surveillance officer (1) |
| **Chris Hani Baragwanath Academic Hospital, University of the Witwatersrand** (6)  South Africa | English | Pediatric oncologist (5)  Oncology fellow (1) |  |  |
| **Guangzhou Women and Children’s Medical Center** (7)  China | Mandarin | Pediatric oncologist (2) Institution officer on COVID-19 (1) | Chief nurse (1) | Volunteer team lead (1) NGO officer (2) |
| **Hospital Saint-Damien/NPFS** (12)  Haiti | French | Pediatric oncologist (2) Emergency room physician (1) Pediatrician (1)  Palliative care (1) Pediatric intensive care (1)  Medical director (1) | Nurse educator (1) Head nurse (1) Infection control nurse coordinator (1) Infection prevention nurse coordinator (1) | Pharmacist (1) |
| **Hospital Martagão Gesteira** (8)  Brazil | Portuguese | Pediatric oncologist (2) Intensive care physician (1) Infectious diseases physician (1) Pediatrician (1) | Nurse director (1) Nurse (1) Infectious diseases nurse (1) |  |
| **Hospital Pediátrico de Sinaloa** (9)  Mexico | Spanish | Pediatric oncologist (4) Anesthesiologist (1) | Nurse (1) | Social Worker (1) Epidemiologist (1) Psycho-oncologist (1) |
| **Instituto Nacional de Enfermedades Neoplásicas (INEN**) (12)  Perú | Spanish | Pediatric oncologist (7) Palliative care physician (1) BMT physician (1) Neuro-oncologist (1) | Chief nurse (1) Nurse (1) |  |
| **Max Super Specialty Hospital** (9)  India | English | Pediatric oncologist (3) Orthopedic surgeon (1) Intensive care physician (1) | Advanced practice nurse (1) | NGO trustee (2) Patient navigator (1) |
| **Philippine General Hospital-** Bedside Providers (7)  Administrative Personnel (3)  Philippines | English | Pediatric oncologist (1) Pediatric oncology fellow (2) | Head nurse (1) | Database manager (1) Child life specialist (1) Patient navigator (1) |
|  |  | Pediatric oncologist (1) Infectious diseases physician (1) Neurosurgeon (1) |  |  |
| **Sardjito General Hospital** (9)  Indonesia | Indonesian | Pediatric oncologist (2) Pediatrician (1) Pediatric intensive care physician (1) Pediatric infectious diseases physician (1) | Nurse (3) | Dietician (1) |
| **St. Jude Children’s Research Hospital**  Bedside Providers (6)  Incident Command Center (8)  USA | English | Pediatric oncologist (2) Intensive care physician (1) | Advanced practice nurse (1) | Dietician (1) Psychologist (1) |
|  |  | Incident commander (1) Lab director (1) Infection control physician (1) | Chief nurse (1) | Patient safety officer (1) Administrator (1) Quality manager (1) Patient experience (1) |
| **The Indus Hospital** (9)  Pakistan | English | Pediatric oncologist (3) Surgeon (1) Infectious diseases physician (1) Executive director (1) | Nurse (1) Infection control nurse lead (1) | Psychologist (1) |
| **Uganda Cancer Institute** (17)  Uganda | English | Pediatric oncologist (3) Radiologist (2) Executive director (1) Pathologist (1) Researcher (1) | Nurse (2) | Hospital administrator (2) Pharmacist (1) Administrator (1) NGO director (2) Researcher (1) |
| **Vall d'Hebron Barcelona Hospital** Group A (15)  Group B (5)  Spain | Spanish | Palliative care pediatrician (1) Anesthesiologist (1) Pediatric surgeon (1) Hematologist (1) | Supervisor nurse (1) Nurse (2) Auxiliary nurse (1) | Foundation representative (1) Clinical trials coordinator (1) Volunteer coordinator (1) Educational psychologist (1) Psycho-oncologist (1) Cytogeneticist (1) Pharmacist (1) |
|  |  | Pediatric oncologist (2) BMT physician (1) Infectious disease physician (1) |  | Social work coordinator (1) |
| TOTAL: 164 Participants  16 Countries | 8 Languages | 88 Physicians | 37 Nurses | 39 Other Roles |
